# Comparison of the Effectiveness and Complications of PAIR, Open Surgery, and Laparoscopic Surgery in the Treatment of Liver Hydatid Cysts

**DOI:** 10.3390/medicina61081351

**Published:** 2025-07-25

**Authors:** Mehmet Sait Berhuni, Veysel Kaya, Hüseyin Yönder, Mehmet Gerger, Mehmet Tahtabaşı, Eyüp Kaya, Hasan Elkan, Faik Tatlı, Ali Uzunköy

**Affiliations:** 1 Faculty of Medicine, General Surgery Department, Harran University, Sanlıurfa 63200, Turkey; hyonder@hotmail.com (H.Y.); mehmetgerger93@gmail.com (M.G.); dr_elkan@hotmail.com (H.E.); faiktatli-@hotmail.com (F.T.); aliuzunkoy@yahoo.com (A.U.); 2 Faculty of Medicine, Radiology Department, Harran University, Sanlıurfa 63200, Turkey; drvkvkfk@gmail.com (V.K.); rddreky@gmail.com (E.K.); 3 Radiology Department, Mehmet Akif İnan Hospital, University of Health Science, Sanlıurfa 63290, Turkey; mehmet.tahtabasi@sbu.edu.tr

**Keywords:** biliary fistula, hydatid cyst surgery, liver hydatid cyst, PAIR, recurrence

## Abstract

*Background and Objectives*: The aim of this study was to compare percutaneous aspiration injection reaspiration (PAIR), open surgery (OS), and laparoscopic surgery (LS) in the treatment of liver hydatid cysts in terms of effectiveness, complications, and recurrence rates. *Materials and Methods*: This retrospective cross-sectional study included 383 patients who were treated with a diagnosis of liver hydatid cyst at Harran University Faculty of Medicine between May 2014 and May 2024. Patients were divided into three groups based on the treatment method: PAIR, OS, and LS. The groups were analyzed in terms of demographic and clinical characteristics such as age, sex, number of cysts, cyst location, and cyst diameter. Various factors such as complications, recurrence rates, and biliary fistula development were compared. Statistical analyses were performed using Jamovi and JASP software, and *p* ≤ 0.05 was considered significant. *Results*: The risk of biliary fistula development was found to be significantly lower in patients treated using PAIR than in those in the surgical groups (*p* < 0.001). While the recurrence rate was higher in the PAIR group, the recurrence rates were similar in the OS and LS groups (*p* = 0.043). The risk of biliary fistula development and catheter removal time were found to be higher in patients with large cysts (*p* < 0.001). A strong and statistically significant correlation was observed between the length of hospital stay and the duration until catheter removal (*p* < 0.001). The maximum diameter of the cyst demonstrated a significant positive correlation with both the length of hospital stay (r = 0.363, *p* < 0.001) and the duration until catheter removal (*p* < 0.001). *Conclusions*: This study demonstrates that the PAIR method is effective in reducing biliary fistula development, but the recurrence rates are higher than OS and LS. OS and LS show similar outcomes in terms of recurrence.

## 1. Introduction

A hydatid cyst is a zoonotic disease caused by the larval stage of flatworms from the genus *Echinococcus*. In humans, it frequently localizes in the liver [1,2]. Although this disease is endemic in regions such as the Mediterranean basin, South America, the Far East, Central Asia, and Eastern Europe, it can also be observed in nonendemic countries due to increasing global travel [3,4,5].

The diagnosis of patients with hydatid cysts is typically based on imaging methods and immunological tests. Most patients with asymptomatic and early-stage hydatid cysts are diagnosed incidentally. Ultrasonography (USG) is a diagnostic tool that can detect the location, number, and size of the cyst relatively easily. When USG is insufficient for diagnosis, computed tomography (CT) and magnetic resonance imaging should be performed [6,7]. Criteria for classifying cysts in the liver were first defined by Gharbi in 1981. Gharbi determined these criteria based on the USG imaging findings of the cysts [8]. These criteria were revised by the World Health Organization (WHO) in 2001 [9].

Four different options are available for treating patients with liver hydatid cysts. While inactive silent cysts may be followed up without any treatment, active cysts can be treated with medical therapy using antihelminthic drugs, surgical intervention, or percutaneous aspiration injection reaspiration (PAIR) [10]. However, evidence-based data on which treatment option should be applied for patients with active cysts remain limited.

Medical treatment is typically used to prevent cyst dissemination, infectivity, and recurrences. In cases with widespread liver involvement or where other treatment options are unsuitable (e.g., patients who are not candidates for surgery or refuse interventional procedures), it is the only treatment option. The drugs used in medical treatment generally belong to the benzimidazole family, and albendazole is the most commonly used drug from this family [11,12].

The aim of surgical treatment in patients is to eradicate the parasite, treat the cyst cavity, manage potential complications, and prevent recurrences [13]. To achieve this, radical (total pericystectomy and lobectomy) or conservative (partial cystectomy) approaches can be employed. Surgical procedures can be performed using open or laparoscopic techniques [14,15,16,17,18].

PAIR is an interventional technique that is considered an alternative to surgery. It can be used for patients with low-stage cysts (WHO Stages 1–2), for those who cannot tolerate surgery, for those who refuse it, or when the cyst’s location within the liver is suitable [2,3,19,20].

The aim of this study was to comparatively evaluate the data of patients who underwent PAIR, open surgery (OS), and laparoscopic surgery (LS) for treating liver hydatid cysts.

## 2. Materials and Methods

Patients who underwent OS and LS for liver hydatid cysts, as well as those who underwent PAIR in the radiology department of our hospital between May 2014 and May 2024, were included in this study. This study was conducted in accordance with the Declaration of Helsinki, and the protocol was approved by the ethics committee of HRÜ (approval number: 24.08.44) on 10 June 2024. 

Postoperative monitoring and outpatient follow-up for patients who underwent both surgery and PAIR were conducted in the general surgery outpatient clinic. Patient data were retrieved from the hospital archive records.

Patients who were under 18 years of age, those with incomplete retrospective records, those with cyst stages other than types I and II based on the WHO-revised hydatid cyst classification (Table 1), those who underwent radical surgery such as pericystectomy or lobectomy, patients presenting with preoperative spontaneous or traumatic rupture, patients undergoing long-term treatment for hydatid cysts, and those with a history of interventional treatment (surgery or PAIR) for hydatid cysts were excluded.

Factors influencing the choice of treatment method included the patient’s preference, the number of cysts, the suitability of the cyst location for the PAIR procedure, and the presence of a high morbidity risk for surgery (American Society of Anesthesiologists IV or higher patients).

For patients with hydatid cysts with a maximum diameter of less than 5 cm, without signs of compression on surrounding tissues, and who were asymptomatic, medical treatment was recommended contingent upon patient consent. Surgery or PAIR was recommended for patients who did not meet any of these three criteria.

In all three methods, the patients were given general anesthesia and intubated before the procedure. PAIR was performed in a fully equipped radiology angiography unit. The anesthesia team took necessary precautions to address any potential anaphylaxis during the procedure.

All patients in the three groups received albendazole treatment (10 mg/kg) for 6 months following the intervention. Patients were called for monthly follow-ups during the treatment period. Complete blood count parameters and liver enzymes were evaluated.

No preprocedural imaging was performed to detect the presence of a bile fistula. Patients who had macroscopic bile-containing fluid oozing from the cyst during or after the procedure were considered to have developed a biliary fistula.

Follow-up outcomes were evaluated according to the WHO-IWGE standardized criteria. Treatment success (healing or inactivation) was defined as the transition of the cyst into a solid or calcified form on follow-up imaging, accompanied by the resolution of symptoms. Recurrence was defined as an increase in the size of the treated cyst or the appearance of new cysts in the liver, other than those previously treated, in a patient considered to have undergone eradication.

Patient data were recorded and evaluated, including age, sex, treatment type, cyst location in the liver lobe, central or peripheral cyst positioning, cyst count, maximum cyst diameter measured by CT or USG, WHO classification stage, presence of intraoperative bile fistula, post-treatment bile drainage from the drain, day to spontaneous resolution of bile leaks, endoscopic retrograde cholangiopancreatography (ERCP) necessity, need for a second catheter due to biloma, presence of major complications (such as perioperative anaphylaxis, cavity infection, recollection, biliary fistula, or intraoperative rupture), length of hospital stay, time to drain removal, recurrence, cyst diameter during follow-up, follow-up period, and mortality rates.

### 2.1. Open Surgery (OS)

A laparotomy was performed through a right subcostal incision, exposing the cyst. After protecting the surrounding tissues with gauze soaked in hypertonic saline, the cyst was punctured using a Veress needle and aspirated. Hypertonic saline was then injected into the cyst, followed by a 10 min waiting period. Thereafter, the cyst was reaspirated. The part of the cyst wall closest to the liver capsule was partially excised using an energy device. The germinal membrane was completely removed. The cyst cavity was irrigated again with hypertonic saline, and bile leakage was checked. For cases where a visible bile leak was detected, an intraoperative repair with 4/0 polypropylene sutures was performed at the leakage site if possible. The operation was completed after placing a drainage catheter in the cyst cavity.

### 2.2. Laparoscopic Surgery (LS)

After inserting a camera port below the umbilicus, additional ports were positioned around the liver according to the cyst location, ensuring good access. Four ports were used along with the camera. The surrounding tissues were protected with gauze soaked in hypertonic saline, similar to OS, and the cyst contents were aspirated via a Veress needle. Hypertonic saline was then injected into the cyst, followed by a 10 min waiting period. Thereafter, the cyst was reaspirated. The part of the cyst wall closest to the liver capsule was partially excised using an energy device. The cyst contents and the excised portion of the cyst wall were removed using an endobag. After irrigating the cyst cavity, the interior was examined for bile leakage using the camera. Any leakage sites were laparoscopically repaired with 4/0 polypropylene sutures. The operation was completed after placing a drainage catheter inside the cyst cavity.

### 2.3. PAIR

In the application of the PAIR procedure, the choice of technique was determined based on whether biliary contamination was detected in the cyst contents. Under USG or fluoroscopic guidance, a needle was inserted into the cyst through a point passing through at least 1 cm of healthy liver tissue. Using USG guidance, an 18-gauge needle was used to puncture the cyst, and 20% of the calculated cyst volume was aspirated. A cystography was performed using 10% contrast agent. Then, 80% of the calculated volume was drained. Once it was confirmed that there was no biliary contamination or fistula to the biliary system, hypertonic saline (30–40% of the initial cyst volume) was injected. After waiting at least 10 min, the contents were aspirated and the needle was withdrawn to terminate the procedure (classic PAIR technique). In cases where macroscopic biliary contamination was observed, the 18-gauge needle was not withdrawn, and a 0.035-inch Amplatz guidewire was advanced into the cyst under fluoroscopy. A 12 French pigtail catheter was inserted and secured to the skin. Once daily drainage fell below 10 cc, the possibility of biliary fistula development was investigated using cystography. If no biliary fistula was detected, ethanol was injected at 30–50% of the initial volume, not exceeding 200 cc in total. After 10 min, the entire cyst content was aspirated. The catheter was removed under fluoroscopy (standard catheterization technique). In patients with persistent biliary fistula on cystography, catheter drainage was continued.

### 2.4. Statistical Analysis

Statistical analyses were performed using Jamovi (Version 2.3.28), JASP (Version 0.19.0), and SPSS (Version 25.0; IBM Corp., Armonk, NY, USA) software. A significance level of *p* ≤ 0.05 was considered statistically significant.

Descriptive statistics were used to summarize the data obtained from this study. Continuous (numerical) variables were presented as the mean ± standard deviation or as median, minimum, and maximum values, depending on the distribution. Categorical variables were summarized as counts and percentages. The normality of numerical variables was assessed using appropriate tests and visual tools based on sample size and data characteristics. For comparisons involving larger samples (*n* ≥ 50), the Kolmogorov–Smirnov and Anderson–Darling tests were employed. Additionally, visual tools such as histograms and Q–Q (quantile–quantile) plots were used to evaluate the assumption of normality.

In cases where heterogeneous distribution was observed between groups, multivariate statistical analyses (logistic and linear regression) were conducted to enhance the internal validity of the comparisons.

For comparisons between two independent groups, the Mann–Whitney U test was used when numerical variables were not normally distributed. In comparisons involving more than two independent groups, the Kruskal–Wallis H test was preferred for non-normally distributed numerical variables. For non-parametric tests, the Dwass–Steel–Critchlow–Fligner test was employed.

In univariate analyses, the effects of variables such as treatment method, number of cysts, cyst localization, maximum cyst diameter, catheter removal time, complications, and other clinical parameters were evaluated individually. The odds ratio (OR), 95% confidence interval (CI), and *p*-values were calculated for each factor. In the multivariate logistic regression analysis, the combined effects of these variables were assessed, independent effects were determined, and OR, 95% CI, and *p*-values were provided for each factor.

## 3. Results

**Figure 1:**
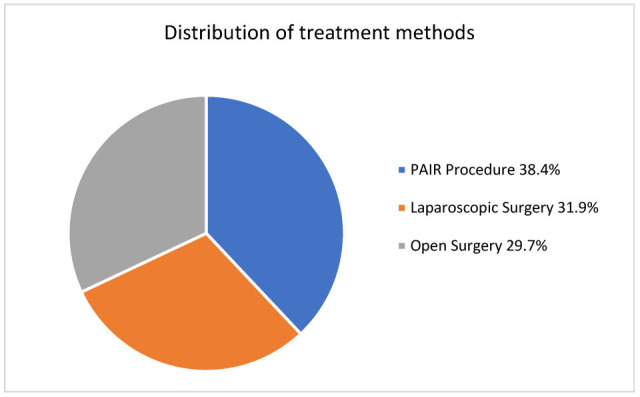
The percentage distribution of the treatment methods used.

This study included 383 patients diagnosed with liver hydatid cysts, and their median age was 33 years. Of the patients, 270 were female (70.5%) and 113 were male (29.5%). Overall, the PAIR method was applied to 38.4% of the patients. LS was performed in 31.9% of the patients (*n* = 122), and OS was applied in 29.7% (*n* = 114) (Figure 1). Demographic and clinical characteristics of the patients, as well as comparisons based on the treatment methods, are presented in Table 2.

The incidence of biliary fistulas detected during PAIR was significantly lower than that during OS and LS (*p* < 0.001). The frequency of bile discharge from the drain after the procedure was highest in the OS group, followed by the LS group, and lowest in the PAIR group (*p* < 0.001). The spontaneous closure rates of biliary fistulas were significantly higher in OS and LS cases than in PAIR cases (*p* < 0.001) (Table 2). ERCP was performed in 47 patients (12.3%) whose biliary fistulas did not close spontaneously. In all of these patients, the fistulas closed following ERCP and subsequent follow-up.

Recurrence was significantly more frequent in patients who underwent PAIR than in those who underwent LS, whereas in patients with OS, it was similar to the other two groups (*p* = 0.043). The follow-up period was significantly shorter in the LS group than in the PAIR and OS groups, and it was also shorter in the OS group than in the PAIR group (LS < OS < PAIR, *p* < 0.001). The need for a second catheter due to bilioma was similar in the PAIR and OS groups and more frequent compared to the LS group (PAIR = OS > LS, *p* = 0.015) (Table 2).

Multivariate logistic regression analysis, adjusting for baseline characteristics including gender, cyst location, cyst localization, WHO stage, intraoperative biliary fistula detection, and treatment modality, demonstrated significant independent associations with clinical outcomes. Open surgery was independently associated with increased risk of biliary fistula formation compared to PAIR (OR: 19.5, 95% CI: 7.8–49.1, *p* < 0.001). Additionally, intraoperative biliary fistula detection markedly increased the postoperative biliary fistula risk (OR: 7.47, 95% CI: 3.26–17.15, *p* < 0.001), whereas peripheral cyst localization showed a protective effect (OR: 0.14, 95% CI: 0.06–0.31, *p* < 0.001). Recurrence rates were significantly lower in peripheral cyst localization (OR: 0.15, 95% CI: 0.04–0.55, *p* = 0.004); however, treatment modality had no independent impact on recurrence. Hospitalization length was independently influenced by cyst localization (central/peripheral, B: +1.84 days, *p* = 0.002) and intraoperative biliary fistula detection (B: −4.13 days, *p* < 0.001), while treatment modality itself was not independently predictive (Table 3).

Recurrence was significantly more frequent in the group treated using PAIR, whereas recurrence rates were similar in the OS and LS cases (*p* = 0.043). Additionally, both the length of hospital stay (*p* = 0.033) and the duration of catheter removal were significantly longer in the recurrence group (*p* = 0.002). The need for a second catheter placement due to bilioma was significantly more common in the recurrence group (*p* = 0.002) (Table 4).

In terms of cyst location, cysts located in both lobes were found to be significantly more common in the biliary fistula group (*p* = 0.001), and there was no difference between the groups in terms of cysts located in the right and left lobes (both *p* > 0.05). Peripheral localization was significantly more common in the group without biliary fistula, and central localization was more common in the group with biliary fistula (*p* < 0.001). The frequency of biliary fistula was similar in both central and peripheral cyst cases. Additionally, in the group with biliary fistula, cyst diameter (*p* < 0.001), length of hospital stay (*p* < 0.001), duration of catheter removal (*p* < 0.001), and cyst size during follow-up (*p* = 0.011) were found to be significantly larger (Table 5).

A strong and significant correlation was found between the length of hospital stay and the duration of catheter removal (r = 0.721, *p* < 0.001). The longest diameter of the cyst showed a significant correlation with both the length of hospital stay (r = 0.363, *p* < 0.001) and the duration of catheter removal (r = 0.328, *p* < 0.001). A weak but significant correlation was also found between the follow-up period and length of hospital stay (r = 0.108, *p* = 0.034, Table 6).

In the univariate analysis, the risk of recurrence was higher in patients who underwent PAIR than in those who underwent LS (OR = 3.11, 95% CI [0.99–9.7], *p* = 0.051), in patients who required a second catheter due to bilioma (OR = 8.55, 95% CI [2.71–27.05], *p* < 0.001), and in cases where the cyst was located in both lobes compared to peripherally located cysts (OR = 5.78, 95% CI [1.68–19.89], *p* = 0.005). However, the risk of recurrence was lower in cases where the biliary fistula closed spontaneously (OR = 0.99, 95% CI [0.03–0.32], *p* < 0.001). The duration of catheter removal was also found to be a significant predictor of recurrence (OR = 1.02, 95% CI [0.99–1.04], *p* = 0.049). Accordingly, a one-unit increase in catheter removal duration increased the probability of recurrence by 2%.

The number of cysts, the longest diameter of the cyst, the occurrence of major complications, the status of biliary fistula, or the localization of the cyst did not significantly affect recurrence (*p* > 0.05 for each).

The treatment method, the number of cysts, location, and cyst localization did not have a significant effect on the risk of major complications (*p* > 0.05 for each).

According to the univariate analysis, the risk of developing biliary fistula was significantly reduced in patients who underwent PAIR compared to those who underwent LS (OR = 0.11, 95% CI [0.06–0.22], *p* < 0.001). The presence of cysts in both lobes compared to cysts in the right lobe (OR = 2.86, 95% CI [1.51–5.44]) and central localization compared to peripheral localization (OR = 5.52, 95% CI [3.16–9.64], *p* < 0.001; *p* = 0.001) were significant factors that increased the risk of biliary fistula. Additionally, an increase in the longest diameter of the cyst (OR = 1.15, 95% CI [1.08–1.23], *p* < 0.001) and the duration of catheter removal (OR = 1.1, 95% CI [1.07–1.14], *p* < 0.001) increased the risk of biliary fistula. Accordingly, a one-unit increase in the longest diameter of the cyst increased the risk of biliary fistula by 15%, whereas a one-unit increase in catheter removal duration increased the risk by 10%.

In the multivariate analysis, the risk of developing biliary fistula was found to be significantly lower in patients who underwent PAIR than in those who underwent LS (OR = 0.04, 95% CI [0.01–0.12], *p* < 0.001). Central localization compared to peripheral localization (OR = 5.56, 95% CI [2.61–11.84], *p* < 0.001) and the longest diameter of the cyst (OR = 1.12, 95% CI [1.02–1.23], *p* = 0.021) continued to be factors increasing the risk of biliary fistula development. The duration of catheter removal also remained significant in the multivariate analysis, increasing the risk of biliary fistula (OR = 1.13, 95% CI [1.08–1.19], *p* < 0.001). No significant effect was observed for the variable indicating the lobe in which the cyst was located (*p* > 0.05).

## 4. Discussion

The present study provides a comprehensive comparison of three treatment modalities—PAIR, laparoscopic surgery (LS), and open surgery (OS)—for liver hydatid cysts, with a particular emphasis on the role of baseline patient characteristics in clinical outcomes. Our findings demonstrate that both the choice of treatment and key patient/cyst features independently influence the risk of complications, recurrence, and hospitalization. 

Consistent with previous reports, we found that PAIR is often utilized in selected subgroups, such as patients with inoperable disease, high surgical risk, or anatomically favorable cysts [2,3,19,20,21]. Thus, direct comparisons between PAIR and surgical modalities must be interpreted in light of inherent differences in patient selection and disease characteristics. Notably, multivariate regression analyses in this study confirmed that treatment modality, cyst localization, intraoperative biliary fistula detection, and cyst size are all significant independent predictors of clinical outcomes. 

Our results revealed that the risk of postoperative biliary fistula was significantly lower following PAIR compared to both LS and OS, even after adjustment for confounding variables (OR: 19.5 for OS vs. PAIR, *p* < 0.001). This aligns with prior meta-analyses and large cohort studies reporting lower complication rates with PAIR relative to surgery [22]. The minimally invasive nature of PAIR, the preservation of cyst wall integrity, and reduced manipulation of biliary structures may underlie these lower rates. Importantly, our findings further indicate that the presence of biliary fistula detected during the procedure and central cyst localization are robust independent risk factors for postoperative biliary complications, corroborating recent evidence [22,23,24,25,26,27]. 

Another finding from the present study is that biliary fistula development was more common in centrally localized cysts, which is also noted in the meta-analysis by Sokouti et al. It was reported that centrally located cysts caused more complications during surgical intervention and that the complication risk was higher in these patients [22]. We believe that the more complex structure of central cysts compared to peripheral ones and their proximity to the bile ducts during surgery may increase these complications.

With respect to recurrence rates, our analysis found these to be highest following PAIR, while both LS and OS were associated with lower recurrence, particularly when peripheral cyst localization was present. These results are in line with previous studies suggesting improved long-term outcomes for surgical approaches in well-selected cases [23,24,25]. However, the lower recurrence seen in OS should be balanced against the higher overall complication risk associated with more invasive procedures.

Hospitalization length was shortest for LS, reflecting the advantages of minimally invasive surgery as described in the previous literature [26,27]. However, our multivariate analysis indicated that the effect of treatment modality on hospital stay was not statistically significant after adjusting for cyst and patient factors, suggesting that the underlying complexity of cysts and perioperative findings are more influential determinants of recovery time than the surgical technique alone.

Of note, larger cyst size and prolonged catheter removal time were both associated with increased risk of biliary fistula and recurrence, supporting prior observations that disease burden and complexity directly impact postoperative outcomes [21,27,28]. These relationships highlight the necessity of individualized treatment planning, particularly in patients with large, centrally located, or complex cysts.

These findings reveal the advantages and limitations of each treatment method, highlighting that treatment decisions should be made according to patient characteristics. Considering parameters such as cyst location and size as well as the risk of biliary fistula development during the follow-up period can help reduce the risk of recurrence. The present study provides significant data on the long-term outcomes of three different methods in the treatment of liver hydatid cysts and offers findings that can guide future treatment approaches.

In summary, our study suggests that the optimal management of liver hydatid cysts requires a nuanced approach that considers both the technical characteristics of the available treatments and the individual patient’s disease profile. Minimally invasive approaches such as PAIR and LS offer advantages in terms of reduced morbidity and hospital stay, but patient selection remains critical, particularly to minimize the risk of recurrence and biliary complications. Future prospective, randomized studies with longer follow-up and more balanced baseline characteristics are needed to refine treatment algorithms and optimize outcomes.

### Limitations

The retrospective nature of this study presents some limitations. Specifically, the retrospective review of patient data resulted in some data not being fully captured due to incomplete or incorrect records. In addition, because this study did not involve randomized patient selection, there may be a significant bias in the distribution of treatment methods. This has made it challenging to assess independent effects on the effectiveness of treatment modalities.

The number of patients among the PAIR, OS, and LS groups was also not equal. The relatively low number of patients treated using PAIR limits the generalizability of the results obtained from this method. Moreover, this study only focused on type 1 and 2 cysts according to the WHO classification, and more advanced-stage cysts were not included in the scope of this study. This limits the generalizability of our findings to more advanced-stage hydatid cyst cases.

A notable limitation of the present study is the significantly higher prevalence of WHO Stage 2 cysts within the OS group, suggesting that this cohort encompassed more advanced or complex cases. This imbalance may have affected the treatment outcomes and complication rates reported for the OS group, thereby posing a constraint on the interpretability of comparisons between treatment modalities. Consequently, the potential impact of disease stage differences on treatment efficacy and complication rates should be thoroughly considered during result interpretation.

Another potential limitation of this study is the relatively short follow-up period of the patients. The mean follow-up duration of 12 months may be insufficient to make comprehensive conclusions regarding recurrence, thereby limiting the ability to generalize long-term outcomes.

## 5. Conclusions

In conclusion, our findings indicate that PAIR is effective in reducing postoperative complications and biliary fistula formation, yet is associated with higher recurrence rates compared to surgical approaches. LS offers advantages in terms of faster recovery and shorter hospitalization, while OS may be preferable in patients with complex or centrally located cysts, albeit with increased risk of complications. The selection of treatment should be individualized, taking into account cyst characteristics (localization, size, stage), patient comorbidities, and intraoperative findings. Further large-scale, prospective studies are required to establish standardized treatment pathways and to better define the long-term efficacy and safety of each modality in diverse patient populations.

## Figures and Tables

**Table 1:** The World Health Organization classification of hydatid cysts.

Stage	Characteristics Findings	Activity
CE1	Unilocular, anechoic cyst with double-line sign	Active
CE2	Multiseptated “rosette-like” “honeycomb pattern” cyst	Active
CE3a	Cyst with detached membrane (water lily sign)	Transitional
CE3b	Daughter cysts in solid matrix	Transitional
CE4	Heterogeneous cyst, no daughter vesicles	Inactive
CE5	Solid matrix with calcified wall	Inactive

CE: cystic echinococcosis.

**Table 2:** Comparisons of demographic and clinical characteristics according to treatment groups in patients with hydatid cysts.

	Overall (*n* = 383)	Treatment Method			*p*
		Laparoscopic (*n* = 122)	PAIR (*n* = 147)	Open (*n* = 114)	
Age	33.0 [18.0–79.0]	34.0 [18.0–79.0]	34.0 [18.0–73.0]	32.0 [18.0–76.0]	0.756 **
Gender					
Female	270 (70.5)	78 (63.9)	114 (77.6) ^b^	78 (68.4) ^a,b^	0.043 *
Male	113 (29.5)	44 (36.1)	33 (22.4) ^b^	36 (31.6) ^a, b^	
Cyst Location					
Right	277 (72.3)	91 (74.6) ^a,b^	113 (76.9) ^b^	73 (64.0)	<0.001 *
Left	61 (15.9)	14 (11.5) ^a^	34 (23.1) ^b^	13 (11.4) ^a^	
Both lobes	45 (11.7)	17 (13.9) ^a^	0 (0.0) ^b^	28 (24.6) ^c^	
Number of Cysts					
Single	268 (70.0)	88 (72.1)	104 (70.7)	76 (66.7)	0.636 *
Multiple	115 (30.0)	34 (27.9)	43 (29.3)	38 (33.3)	
Localization					
Peripheral	295 (77.0)	89 (73.0)	112 (76.2)	94 (82.5)	<0.001 *
Central	69 (18.0)	32 (26.2) ^a^	18 (12.2) ^b^	19 (16.7) ^a,b^	
Both	19 (5.0)	1 (0.8) ^a^	17 (11.6) ^b^	1 (0.9) ^a^	
Longest Diameter of the Cyst (mm)	9.0 [3.0–23.0]	9.0 [4.0–21.0]	9.0 [3.0–19.0]	9.5 [5.0–23.0]	0.553 **
WHO Stage					
Stage 1	242 (63.2)	90 (73.8)	110 (74.8)	42 (36.8) ^b^	<0.001 *
Stage 2	141 (36.8)	32 (26.2) ^a^	37 (25.2)	72 (63.2) ^b^	
Biliary Fistula Detected During the Procedure	116 (30.3)	54 (44.3) ^a^	12 (8.2) ^b^	50 (43.9)	<0.001 *
Biliary Discharge from Drain After the Procedure	229 (59.8)	89 (73.0)	40 (27.2) ^b^	100 (87.7) ^c^	<0.001 *
Spontaneous Closure of Biliary Fistula	182 (79.5)	79 (88.8) ^a^	19 (47.5) ^b^	84 (84.0) ^a^	<0.001 *
ERCP for Biliary Fistula	47 (12.3)	10 (8.2)	21 (14.3)	16 (14.0)	0.251 *
Major Complication	69 (18.0)	26 (21.3)	22 (15.0)	21 (18.4)	0.399 *
Periop Anaphylaxis	16 (4.2)	10 (8.2) ^a^	3 (2.0) ^b^	3 (2.6) ^a,b^	0.043 *
Cavity Infection	26 (6.8)	6 (4.9)	12 (8.2)	8 (7.0)	0.570 *
Recollection	1 (0.3)	0 (0.0)	0 (0.0)	1 (0.9)	0.295 *
Sign of Intraop Cyst Rupture	8 (2.1)	5 (4.1) ^a^	0 (0.0) ^b^	3 (2.6) ^a^	0.038 *
Length of Hospital Stay (Days)	6.0 [1.0–57.0]	5.0 [2.0–57.0]	6.0 [1.0–50.0]	6.0 [2.0–25.0]	0.065 **
Catheter Removal Duration (Days)	6.0 [1.0–270.0]	5.0 [1.0–48.0]	7.0 [1.0–270.0]	6.0 [2.0–35.0]	0.093 **
Recurrence	22 (5.7)	4 (3.3) ^a^	14 (9.5) ^b^	4 (3.5) ^a,b^	0.043 *
The first imaging modality used in patient follow-up					
CT	236 (61.6)	122 (100.0) ^a^	0 (0.0) ^b^	114 (100.0) ^a^	<0.001 *
USG	147 (38.4)	0 (0.0) ^a^	147 (100.0) ^b^	0 (0.0) ^a^	
Cyst Dimension During Follow-Up (Longest Diameter—mm)	5.0 [2.0–17.0]	6.0 [2.0–17.0]	5.0 [2.0–12.0]	5.0 [2.0–17.0]	0.066 **
Follow-Up Period (Month)	12.0 [1.0–60.0]	6.0 [1.0–48.0]	12.0 [6.0–60.0]	12.0 [1.0–60.0]	<0.001 **
Mortality					
Surviving	379 (99.0)	121 (99.2)	146 (99.3)	112 (98.2)	0.693 *
Ex	4 (1.0)	1 (0.8)	1 (0.7)	2 (1.8)	
Second Catheter Insertion Due to Bilioma	17 (4.4)	0 (0.0) ^a^	9 (6.1) ^b^	8 (7.0) ^b^	0.015 *

^a^, ^b^, ^c^: Different superscripts indicate statistical differences between groups in each row. There is no statistical difference between groups with the same superscripts. *. Kruskal–Wallis H test. **. Pearson chi-square, Fisher–Freeman–Halton test.

**Table 3:** Results of multivariate regression analyses for primary clinical outcomes (biliary fistula formation, recurrence, and hospitalization length), adjusted for all significant baseline patient characteristics including treatment modality, gender, cyst location and localization, WHO stage, and intraoperative findings.

Outcome	Independent Variable	OR or B (95% CI)	*p*-Value	Interpretation
Biliary Fistula Formation	Treatment Modality (Open vs. PAIR)	OR: 19.5 (7.8–49.1)	<0.001	Significantly higher risk in open surgery
	Treatment Modality (Laparoscopic vs. PAIR)	OR: 1.08 (0.60–1.94)	0.80	Not significant
	Intraoperative Biliary Fistula	OR: 7.47 (3.26–17.15)	<0.001	Strong independent risk factor
	Cyst Localization (Peripheral vs. Central)	OR: 0.14 (0.06–0.31)	<0.001	Protective
	WHO Stage II vs. I	OR: 0.49 (0.23–1.04)	0.06	Trend, not significant
Recurrence	Cyst Localization (Peripheral vs. Central)	OR: 0.15 (0.04–0.55)	0.004	Significantly lower recurrence in this localization
	Treatment Modality	—	NS	No independent effect
	Other variables	—	NS	—
Hospitalization Length	Cyst Localization (Peripheral vs. Central)	B: +1.84 (±0.60)	0.002	Increases length of stay
	Intraoperative Biliary Fistula	B: −4.13 (±0.73)	<0.001	Decreases length of stay
	Treatment Modality	—	NS	No independent effect

OR, odds ratio; CI, confidence interval; B, regression coefficient; NS, not significant.

**Table 4:** Comparisons of demographic and clinical characteristics according to recurrence status in patients with hydatid cysts.

	Recurrence Status		*p*
	Yes (*n* = 22)	No (*n* = 361)	
Treatment Method			
LS	4 (18.2) ^a^	118 (32.7)	0.043 *
PAIR	14 (63.6)	133 (36.8) ^b^	
OS	4 (18.2) ^a^	110 (30.5) ^a^	
Localization			
Peripheral	13 (59.1) ^a^	282 (78.1) ^b^	0.013 *
Central	5 (22.7) ^a^	64 (17.7)	
Both	4 (18.2) ^a^	15 (4.2) ^b^	
Spontaneous Closure of Biliary Fistula	5 (33.3)	177 (82.7)	<0.001 *
ERCP for Biliary Fistula	10 (45.5)	37 (10.2)	<0.001 *
Length of Hospital Stay (Days)	8.0 [1.0–50.0]	5.0 [1.0–57.0]	0.033 **
Catheter Removal Duration (Days)	17.5 [1.0–100.0]	6.0 [1.0–270.0]	0.002 **
The First Imaging Modality Used in Patient Follow-Up			
CT	8 (36.4)	228 (63.2)	0.022 *
USG	14 (63.6)	133 (36.8)	
Second Catheter Insertion due to Bilioma	5 (22.7)	12 (3.3)	0.002 *

a, b: Different superscripts indicate statistical differences between groups in each row. There is no statistical difference between groups with the same superscripts. *. Mann–Whitney U test. **. Pearson chi-square, Fisher’s Exact/Fisher–Freeman–Halton test.

**Table 5:** Demographic and clinical characteristics in hydatid cyst cases and comparisons according to the presence of biliary fistula.

	Biliary Fistula		*p*
	Yes (*n* = 116)	No (*n* = 267)	
Treatment Method			
LS	54 (46.6)	68 (25.5) ^b^	<0.001 *
PAIR	12 (10.3) ^a^	135 (50.6) ^b^	
OS	50 (43.1)	64 (24.0) ^b^	
Cyst Location			
Right	79 (68.1) ^a^	198 (74.2)	0.001 *
Left	13 (11.2) ^a^	48 (18.0) ^a^	
Both lobes	24 (20.7)	21 (7.9) ^b^	
Localization			
Peripheral	68 (58.6)	227 (85.0) ^b^	<0.001 *
Central	43 (37.1)	26 (9.7) ^b^	
Both	5 (4.3) ^a^	14 (5.2) ^a^	
Longest Diameter (mm)	10.0 [5.0–23.0]	9.0 [3.0–20.0]	<0.001 **
Length of Hospital Stay (Days)	7.0 [2.0–57.0]	5.0 [1.0–34.0]	<0.001 **
Catheter Removal Duration (Days)	10.0 [1.0–270.0]	5.0 [1.0–60.0]	<0.001 **
Cyst Dimension During Follow-Up (Longest Diameter—mm)	6.0 [2.0–15.0]	5.0 [2.0–17.0]	0.011 **

a, b: Different superscripts indicate statistical differences between groups in each row. There is no statistical difference between groups with the same superscripts. *. Mann–Whitney U test. **. Pearson chi-square test.

**Table 6:** Correlations between length of hospital stay, duration of catheter removal, age, and cyst characteristics.

	Length of Hospital Stay (Days)		Catheter Removal Duration (Days)	
	r	*p*	r	*p*
Catheter Removal Duration (Days)	0.721	<0.001		
Age	0.106	0.038	0.059	0.252
Longest Diameter (mm)	0.363	<0.001	0.328	<0.001
Follow-Up Period (Months)	0.108	0.034	0.083	0.103

Spearman’s rho correlation coefficient was used.

## Data Availability

The original contributions presented in this study are included in the article. Further inquiries can be directed to the corresponding author.
